# Teaching psychiatry to medical students in the time of COVID-19: experiences from UK medical schools

**DOI:** 10.1192/bjb.2021.67

**Published:** 2021-07-27

**Authors:** Ho Tim Timothy Leung, Ali Ajaz, Helen Bruce, Ania Korszun

**Affiliations:** 1 East London NHS Foundation Trust, UK; 2 Wolfson Institute of Preventive Medicine, Queen Mary University of London, UK

**Keywords:** Education and training, psychiatry, COVID-19, medical students, adaptation

## Abstract

**Aims and method:**

Education leads for undergraduate psychiatry in UK medical schools completed questionnaires on adaptations made to undergraduate psychiatry education, their impact and what lessons could be learnt for the future.

**Results:**

Respondents from 24 medical schools across the UK reported a major shift to online teaching delivery, with reduced workplace learning and increased use of teleconferencing, online tasks and self-directed learning. Changes were implemented with some faculty training provided, but little additional funding or resources from medical schools or National Health Service trusts. A variety of challenges and opportunities were reported.

**Clinical implications:**

Despite the extraordinary efforts of education leads to maintain undergraduate psychiatry education, the pandemic may affect the development of students’ professional competencies and recruitment into psychiatry. Individual clinicians, trusts and medical and foundation schools have much to offer, and need to work with students to replace what has been lost during the pandemic.

In the UK, the first lockdown in response to the COVID-19 pandemic was announced on 23 March 2020. This prompted rapid changes in medical education, including withdrawal of students from clinical placements and a shift to online teaching. As the pandemic continues, undergraduate medical educators face difficult choices to ensure a continuing supply of competent medical professionals for the future healthcare system.^1^ Clinical skills in psychiatry will particularly be needed to address the significant impact of the pandemic on mental health. Increased depression and anxiety symptoms have been reported, including in young people,^2–4^ and long-term consequences are emerging in those who have recovered from COVID-19.^5^ Furthermore, many healthcare professionals are themselves experiencing symptoms of anxiety spectrum disorders.^6,7^

To prepare our future doctors for the expected rise in demand, high-quality psychiatric education must not only continue, but also be prioritised. There is no consensus or guidance yet on what and how adaptations to undergraduate psychiatry teaching should be implemented during the pandemic. Although individual institutions outside of the UK have shared their experiences,^8–11^ there has been no work published to date from the UK or on the collective experiences of different institutions. Understanding the experiences of multiple institutions provides a powerful opportunity to share learning and identify the needs of this cohort of students.^12^

## Objectives

Our aims were to identify what and how adaptations to undergraduate psychiatry teaching were made in response to the pandemic restrictions, their impact and what lessons could be learnt from the collective experiences of UK medical schools.

## Method

We conducted an online cross-sectional questionnaire survey of education leads for undergraduate psychiatry who represent their medical schools on the Royal College of Psychiatrists Undergraduate Education Forum (UEF). The questionnaire was drafted on Microsoft Forms (Microsoft, Richmond, Washington, U.S.; see https://forms.microsoft.com/). Questions were based on areas of adaptations identified in Gordon et al's scoping review^13^ and current reports of adaptations in psychiatry teaching from other countries.^8–10^ The UEF contributed to the final design of the questionnaire, which consisted of both open and closed questions, and included the following themes: changes to teaching delivery and content, changes to the psychiatry rotation, changes to assessment, support for learners, faculty development, funding and resources, and impact of adaptations.

Respondents were asked to consider adaptations in the current academic year. As clinical placements in psychiatry in most schools occur in the penultimate year, responses were included only from the 33 registered and publicly funded UK medical schools established before 2018.^14^ The Queen Mary Ethics of Research Committee advised that as an evaluation of teaching, research ethics review was not required. The full survey is available in Supplementary File 1 available at https://doi.org/10.1192/bjb.2021.67.

The survey was distributed via email to the members of the UEF on 20 November 2020, with one reminder email a month later. It remained open for 2 months and participation was voluntary. Where omissions were present in returned questionnaires, respondents were contacted for clarification.

Data were exported from Microsoft Forms to Microsoft Excel version 16.33 for Mac, which was used to calculate descriptive statistics and create graphs. Open-ended text responses were reviewed to describe adaptations in more detail, and to compile a summary of opportunities and challenges for psychiatry teaching during the pandemic.

## Results

### Respondents

A total of 24 education leads completed the survey (72.7% response rate). Respondents from schools in all four nations of the UK participated, with 19 from England, 2 from Wales, 2 from Scotland and 1 from Northern Ireland.

### Moving to online delivery

**Fig. 1:**
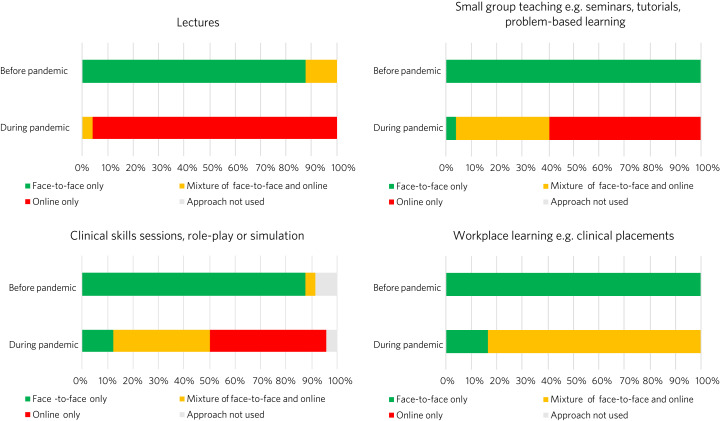
Method of delivery of different teaching approaches before and during the pandemic.

Figure 1 shows the changes that were made to delivery of different teaching approaches, with a major shift to online delivery.

Schools differed widely in the percentage of online teaching delivered synchronously, with an average of 66.1% and range of 10–100% (*n* = 22). In the 23 schools where it was used, asynchronous teaching employed a range of existing resources, primarily e-learning resources (82.6%) and recordings of previous content (82.6%) from respondents’ own medical schools and e-learning resources obtained through the UEF (73.9%). Of the schools that used asynchronous teaching, 87% created new resources for this purpose. These included new recordings of teaching sessions, simulated patient videos, online modules, workbooks to consolidate materials and guide learning, revision notes, quizzes, question banks and serious games.

### Course content

A total of 83.3% of schools reported no change in the content of the psychiatry curriculum; in three of the four schools where a change was made, these had already been planned before the pandemic, and were implemented during the pandemic or brought forward.

**Fig. 2:**
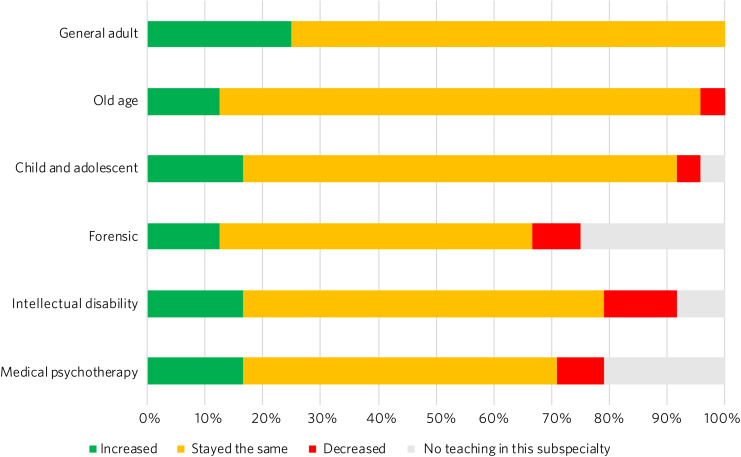
Responses to the question ‘Has the amount of teaching in the following subspecialties increased, decreased or stayed the same?’.

The amount of teaching across the six psychiatric subspecialties stayed the same in the majority of schools (Fig. 2). For every subspecialty, more schools increased rather than decreased teaching; 25% of schools increased teaching in general adult psychiatry, with no schools decreasing teaching in this subspecialty. The subspecialties that had most decreases in teaching were psychiatry of intellectual disabilities (12.5%), forensic psychiatry (8.3%) and medical psychotherapy (8.3%).

### Learning approaches

**Fig. 3:**
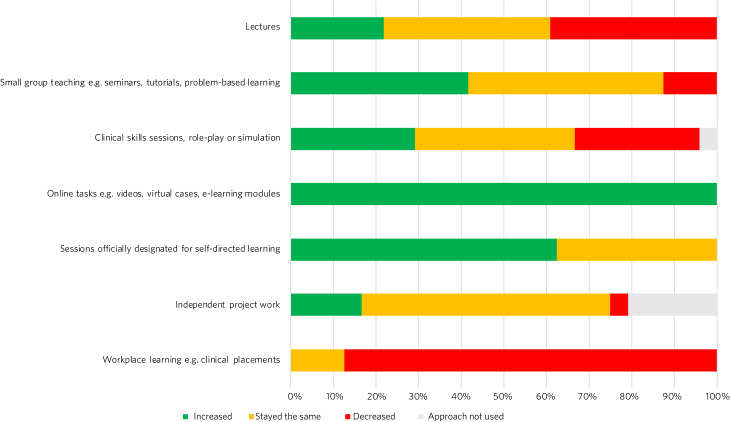
Responses to the question ‘Has the proportion of time spent by students learning using the following approaches increased, decreased or stayed the same?’.

Although 66.7% of schools made no change to the length of the overall psychiatry module, 12.5% increased and 20.8% reduced the length. Also, 66.7% of schools did not change the length of clinical placements; however, respondents noted that in practice, there was reduced time in clinical settings and more time in self-directed learning. This is reflected in 87.5% of schools decreasing the proportion of time students spent in workplace learning (Fig. 3). In addition, 33.3% of schools decreased the length of clinical placements. All schools increased the proportion of time students spent completing online tasks, and 62.5% of schools increased sessions officially designated for self-directed learning. More schools increased small group teaching (41.7%) than lectures (21.7%).

### Clinical placements

**Fig. 4:**
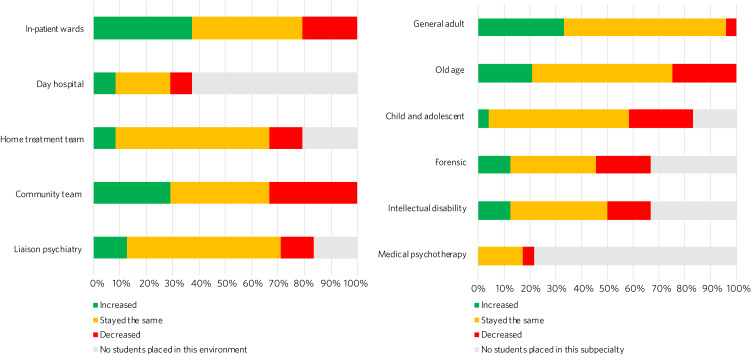
Responses to the question ‘In the current academic year, have the number of students placed in the following clinical environments/subspecialties increased, decreased or stayed the same?’.

A total of 37.5% of schools increased numbers of students placed in in-patient wards, and 33.3% of schools decreased numbers placed in community teams (Fig. 4). For most schools, numbers of students placed in home treatment teams (58.3%) and liaison psychiatry (58.3%) stayed the same.

Most schools placed the same number of students in general adult psychiatry (62.5%), old age psychiatry (54.2%) and child and adolescent psychiatry (54.2%) during the pandemic (Fig. 4). General adult psychiatry had the most schools increasing numbers of students placed in the subspecialty (25%), whereas old age psychiatry (25%) and child and adolescent psychiatry (25%) had the most schools decreasing numbers placed.

### Teleconferencing in clinical placements

A total of 75% of schools had teleconferencing in some clinical placements, with 20.8% in all placements. The only school where there was no teleconferencing had moved all placements to the in-patient setting. Teleconferencing allowed students to engage in clinical activities remotely from home or from the same location as the clinician. Students observed and participated in out-patient reviews, and joined meetings and ward rounds. One school recruited patients who could be interviewed by students via teleconferencing.

### Assessment

Regarding assessment, 66.7% of schools reported that assessment of the psychiatry rotation changed during the pandemic. Changes included reduced demands, such as reduced emphasis on documenting experiences, reduced numbers of workplace-based assessments or case presentations, and reduced attendance monitoring. Despite reduced assessment demands, respondents were clear that standards would not be lowered or compromised.

Some schools shifted toward formative assessments; for example, using an assessed clinic letter or a portfolio of cases, reflections and workplace-based assessments rather than a clinician-observed long case. Greater onus was placed on individual supervisors to oversee satisfactory student performance. Like teaching delivery, assessment moved online. Online platforms, video stations and virtual cases were used to assess clinical skills, case-based discussions and presentations, and logbooks were completed online.

### Supporting learners

In 41.7% of schools, psychiatry teachers were involved in developing resources or interventions to support learners’ mental health and well-being during the pandemic. Respondents described developing well-being talks and workshops. They established well-being drop-ins, well-being champions, virtual student messes, and pastoral and reflective groups, including for students volunteering in the National Health Service (NHS). Well-being information was provided through newsletters and online platforms. Existing mechanisms to support learners were bolstered or reiterated to students.

### Faculty development

In 50% of schools, faculty received some training in adapting teaching in response to COVID-19, with training in online teaching provided in 58.3% of schools. Training led by medical schools included sessions or e-learning modules on delivering remote learning (e.g. blended learning design, platforms), access to e-learning authoring tools, individual discussions with education leads and opportunities to share practice. Such training was less accessible to clinical staff, who received additional support from psychiatry education leads. This included training on online platforms, reflective groups to share practice and ensuring adequate technology at clinical sites.

### Funding and resources

A total of 95.8% of education leads received no additional funding or resources from their medical school to deliver psychiatry teaching during the pandemic; only one school reported such support, which was additional funding to develop e-learning materials. Further, 79.2% of education leads received no additional funding or resources from their affiliated NHS trusts. In those trusts that provided additional support, this included laptops for students, computer equipment, funding for consultant psychiatrist time to coordinate placements and support clinicians, and funding for actors or patients to be interviewed by students practising clinical skills.

### Student involvement and response

**Fig. 5:**
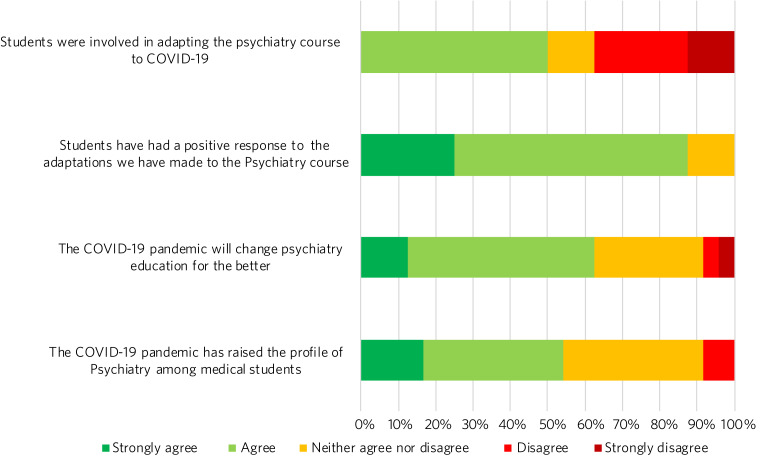
Responses to the question ‘To what extent do you agree or disagree with the following statements?’.

Half (50%) of education leads agreed that students were involved in adapting the psychiatry course (Fig. 5). Education leads reported that students had responded positively to adaptations, with 87.5% agreeing or strongly agreeing with the statement.

### The future

A total of 62.5% of respondents agreed or strongly agreed that the pandemic will change psychiatry education for the better (Fig. 5), and 54.2% of respondents agreed or strongly agreed that the pandemic has raised the profile of psychiatry among medical students.

In 87.5% of schools, the psychiatry rotation will not return to exactly how it was before the pandemic. Changes will include increased blended learning, with respondents noting the advantages of facilitating access from distant placements and the ability to accommodate increases in student numbers. Other changes include keeping modifications in placement structure, online logbooks and the use of resources from other schools.

## Discussion

This survey of adaptations to psychiatry teaching from 24 medical schools across the four nations of the UK demonstrates the seismic impact of the pandemic on psychiatry education. Table 1 summarises both the opportunities and challenges that emerge from the survey data, with proposed solutions to meet the challenges.

**Table 1:** Opportunities, challenges and proposed solutions for undergraduate psychiatry education during the pandemic

	Opportunities	Challenges	Proposed solutions
Students	Accessibility and flexibility of online delivery Reduced travel Asynchronous teaching accessible at convenient times Clinical experiences available remotely, including for self-isolating students Gaining familiarity with telemedicine Educational advantages of blended learning and flipped classrooms	Requirements needed for online delivery Adequate bandwidth and hardware Limited access to technology in clinical environments Time spent learning new technology Experiences of online delivery ‘Zoom fatigue’ Negative impact of isolation on student well-being Less engaging than face-to-face delivery Difficulty in engaging with self-directed learning Clinical placements Reduced contact with patients, so reduced opportunities to develop clinical skills In in-patient settings, space constraints and need for social distancing may prevent students from accessing opportunities such as ward rounds in person In community settings, there are fewer opportunities to engage with patients face to face and learn to be in the room with patients Travel to face-to-face placements during pandemic Service changes resulting in last-minute cancellations and timetabling changes Difficult to get signed off by busy clinicians Experiences of teleconferencing on clinical placement Less engaging than face to face Juggling multiple IT accounts More difficult for students to participate in clinical tasks Missing out on pre-brief and debrief before and after consultations Hard to feel part of the team remotely	Online delivery Investment in dedicated educational facilities at hospital sites Scheduling adequate breaks Interventions to address student well-being Systems to identify students who are engaging poorly with online learning Educating students about how to approach online and self-directed learning Clinical placements Inclusion of training in psychiatry in foundation programmes and beyond Increased clinical and communication skills training delivered outside of clinical placements Honest dialogue with students about the potential for disruption to their learning during the pandemic Making use of all available opportunities for face-to-face contact with patients, e.g. students shadowing on-call staff Pairing in-patient and community placements Ring-fenced time for undergraduate education in clinicians’ job plans Teleconferencing on clinical placement Training teachers to include student participation in consultations Deliberate inclusion of pre-brief and debrief time Including students in all team activities, e.g. meetings Including multidisciplinary team members in teaching
Teachers	Accessibility of online delivery Gaining familiarity with telemedicine Opportunities for interactivity in online delivery	Increased clinical pressures Online delivery Time spent learning new technology with little training Some students engage less with synchronous teaching, e.g. cameras switched off Managing unprofessional behaviours Teleconferencing Logistical demands of setting up teleconferencing Difficult to build teacher–student relationships remotely	Increased training on online delivery Setting of expectations for student engagement with teaching and digital professionalism Promoting continuity to encourage the building of teacher–student relationships. e.g. same tutor throughout placement Ring-fenced time for undergraduate education in clinicians’ job plans
Course content	Accessibility of online delivery facilitates Webinars with external speakers Attendance at mental health tribunals Increased involvement of experts by experience Increased teaching in some subspecialties Asynchronous teaching Expanded offer available to students Standardisation of teaching quality	Service changes (e.g. ward closures) limit some learning opportunities Access to other learning opportunities (tasters) not possible because of social distancing	Increased online teaching on certain areas to compensate for lost learning opportunities Inclusion of training in psychiatry in foundation programmes and beyond
Course organisation	Booking and availability of rooms no longer of concern Same lectures do not need to be recorded multiple times More immediate student feedback leading to rapid improvement of quality Exposing underfunding in undergraduate education to justify additional resources Showing that online learning can be a solution to accommodating increases in student numbers Increased collaboration between schools, e.g. sharing resources	Some clinicians are less keen to host students and engage in teleconferencing Need for faculty development for online teaching Increased administrative burden of organising online delivery and redesigning clinical placements in accordance with public health measures, e.g. staff working from home, social distancing and bubbles	Incentives for clinicians to contribute to undergraduate teaching Increased training on online delivery Increased administrative support and resources from medical schools and National Health Service trusts
Promoting psychiatry	Improved attendance by students Increased recognition of the importance of reflective practice across all specialties Increased focus on student and staff well-being Showcasing psychiatry as an exemplar of innovations in adapting teaching	Reduced opportunities to meet role models with less time on clinical placement Reduced opportunities to combat stigma toward mental illness with reduced contact with patients	Maximising learning opportunities during shortened clinical placements Increased activity of student-led psychiatry societies to promote psychiatry Building in opportunities to meet psychiatrists and experts by experience Implementing specific training on stigma toward mental illness Increased postgraduate training in psychiatry clinical skills

### Developing clinical skills in psychiatry

The Royal College of Psychiatrists’ curriculum recommendations, informed by the General Medical Council's (GMC) ‘Outcomes for Graduates’,^15^ states that an important aim of undergraduate psychiatry education is for ‘students to develop the necessary skills to apply [professional] knowledge in clinical situations’.^16^

Clinical placements form the bulk of students’ experience in psychiatry in the UK,^17^ offering opportunities for experiential learning and participation in authentic clinical environments.^18^ However, 87.5% of schools were forced to decrease the proportion of time that students spent on clinical placement. Even when clinical placements were possible, service changes and social distancing requirements changed the nature of their learning opportunities. Without these experiences, students may have difficulty in understanding how to apply their professional knowledge in clinical contexts. Indeed, the Medical Schools Council notes that ‘it is not possible for students to meet the requirements set out in the GMC's Outcomes for Graduates without undertaking clinical placements’.^19^

During the pandemic, schools continued to provide clinical skills, role-play or simulation teaching, with 45.8% delivering these fully online. Although online skills teaching can alleviate students’ concerns about reduced patient contact,^20^ learners feel less prepared to use skills learnt in practice.^21^ In a survey of UK medical students in May 2020, three-quarters felt that online teaching had not successfully replaced the clinical teaching that they received from direct patient contact.^22^

A total of 95.8% of schools used teleconferencing on clinical placements, which, though useful, has limitations. For instance, the court judgment on remote Mental Health Act assessments noted that ‘a psychiatric assessment may often depend on much more than simply listening to what the patient says … [and] may involve a multi-sensory assessment’.^23^ In consultations by teleconferencing, clinicians face difficulties in reading non-verbal communication, using silence and incorporating physical examination.^24^ Without the opportunity to see clinicians demonstrating these skills and to practise these skills themselves, students are left with an experience that translates poorly to the face-to-face situations they will encounter in the future. Moreover, clinicians cannot model some skills that are important in face-to-face work, such as preparing consultation rooms or judging physical distances between patient and clinician. Nevertheless, telepsychiatry is likely to be used more widely in the future.^25^ Early training can foster specific skills, such as conducting mental state examinations by telephone.^26^ These should supplement, but not supplant, the acquisition of skills for face-to-face interactions.

The shift away from workplace learning was accompanied by an increase in self-directed learning and the use of online tasks. Self-directed learning prepares students for lifelong learning, and online tasks provide the opportunity to develop a broader knowledge base. However, some schools decreased teaching in the subspecialties, with psychiatry of intellectual disabilities, forensic psychiatry and medical psychotherapy most affected. This means that the only available opportunities to learn skills in these subspecialties may have been lost; for example, learning to communicate with people with intellectual disabilities and understanding unconscious aspects of the doctor–patient relationship.

The fact that assessments have continued with no change in standards during the pandemic is reassuring. Indeed, the greater emphasis on formative assessments and developmental conversations with individual clinicians may provide more opportunities for students to receive feedback.

### Attitudes toward mental illness

Reductions in time spent in clinical placements mean that students get less contact with people with psychiatric conditions, which is so important in dispelling stigma toward mental illness.^27^ The relative shift away from placements in community teams toward in-patient wards during the pandemic may also have unintended consequences; in a meta-analysis conducted before the pandemic, in-patient placements had less effect in challenging stigmatising attitudes than community or mixed placements.^28^

On the other hand, the greater emphasis placed on the mental well-being of students^29^ and healthcare staff^30^ during the pandemic may encourage students to pay attention to their own health and well-being, and raise their awareness of the importance of mental health. Psychiatry teachers are particularly well-equipped, with expertise in both mental health and undergraduate education, to support students.

### Recruitment into psychiatry

Experiences during clinical placements affect career choices, with just over half of students reporting that they were more inclined to choose a career in psychiatry following their placement.^31^ Placement factors that encourage students to choose psychiatry include perceived clinical responsibility and influence of teachers as role models.^32,33^ Although an international survey found no relationship between placement length and choosing psychiatry,^32^ a placement should be sufficiently long for students to get involved in the team and follow patients’ progress.^34^ Shifting away from workplace learning reduces such opportunities and may affect recruitment into psychiatry.

The pandemic has also limited opportunities (e.g. through lack of availability of electives) for fully exploring different subspecialties.^35^ Tasters, where students spend short periods of time experiencing subspecialties outside of their main clinical placement, demonstrate to students the breadth of opportunities that a career in psychiatry entails.^36^ Social distancing measures limit access to tasters. Despite these limitations, most education leads agreed that the pandemic had raised the profile of psychiatry among medical students. Increased awareness of reflective practice and a renewed focus on student well-being may have contributed to this. Whether this will translate into more positive attitudes toward psychiatry from other specialties is unclear.^37^

### A call to action in a time of change

The pandemic has been a catalyst for spurring innovations in medical education.^38^ Even after the pandemic, there will be changes to psychiatry teaching in the majority of schools. Most education leads are optimistic that the pandemic will change psychiatry education for the better. Students have responded positively to the adaptations to teaching and, mirroring the changes predicted by our respondents, want to continue with online lectures and increased access to online resources in the future.^39^ It remains to be seen whether reactive adaptations implemented during the pandemic will be sustainable, and help to manage another impending challenge: the surge in student numbers resulting from the government temporarily lifting the cap on medical school places.^40^

One year on from the first national lockdown, the course of the pandemic remains uncertain. But what is becoming more certain is the profound impact that the pandemic has had on the way we teach psychiatry. This is most marked in the reduction of clinical placements, which may affect the attainment of key competencies relevant to practice in all branches of the profession, exposure to clinical role models and the challenging of stigmatising attitudes to mental illness and psychiatry. Reduction in clinical placement may also pose a threat to future recruitment into psychiatry at a time when there will be an even greater need for psychiatric skills, to manage increased demand following the pandemic.^41,42^

This is the time for all of us in psychiatry to unite and rise to the challenges that lie ahead. We call upon individual clinicians, NHS trusts and medical and foundation schools to work closely together and with students, to give this generation the training that they need to best care for patients in the post-pandemic landscape.

Individual clinicians can offer so much to maximise the quality of learning during shortened placements. They can offer opportunities for students to participate fully in the care of patients and foster experiential learning. Trusts continue to receive payment for educating students, even with reduced time spent in clinical settings, and can ensure a more equitable distribution of finances to front-line educators.^43^ Teaching during the pandemic has required considerable planning and effort from education leads, yet in our survey, few reported receiving additional funding or resources.

Medical schools should incorporate opportunities, wherever possible, for students to meet psychiatrists and experts by experience, to implement training on stigma and support the activities of student-led psychiatry societies.^44^ Lost learning opportunities should be prioritised for inclusion in online teaching programmes both during and beyond medical school. Foundation schools should similarly increase postgraduate psychiatry teaching and the number of posts in psychiatry. Greater collaboration between foundation programme leads and undergraduate educators is important to replace what has been lost during the pandemic.

Undergraduate psychiatry teaching in the UK has undergone extensive adaptations in response to COVID-19. Educators have done a remarkable job to maintain the integrity of teaching in the face of considerable adversity. Lessons learnt during the pandemic will shape teaching in the future. As we start our journey into the post-pandemic world, we hope that readers will heed our call to action. We must not let the pandemic set back decades of progress in the care of people with mental illness.

### Strengths and limitations

To our knowledge, this is the first nationwide survey of adaptations in undergraduate psychiatry teaching in any country. There was a good response rate of 72.7%, with responses from all four nations of the UK. By asking education leads about specific areas of adaptations, we may not have captured all the adaptations made at individual schools. Similarly, there was variable completion of open questions and depth in respondents’ descriptions of adaptations. As a survey of education leads, we have not explored first-hand the views of students and other clinicians on the impact of adaptations. Lastly, the survey offers a snapshot of adaptations at a particular moment in time, and does not capture longitudinal changes in response to the course of the pandemic.

## About the authors

**Ho Tim Timothy Leung** is a Fellow in Medical Education in the Medical Education Department at East London NHS Foundation Trust, UK. **Ali Ajaz** is a Consultant Psychiatrist in the Forensic Mental Health Service at East London NHS Foundation Trust, UK. **Helen Bruce** is a Consultant Psychiatrist in Child and Adolescent Mental Health Services and Associate Dean for Undergraduate Education at East London NHS Foundation Trust, UK. **Ania Korszun** is Professor of Psychiatry and Education at the Wolfson Institute of Preventive Medicine, Queen Mary University of London, UK.

## Data Availability

The data that support the findings of this study are available from the corresponding author, A.K., upon reasonable request.

## Supplementary material

For supplementary material accompanying this paper visit https://doi.org/10.1192/bjb.2021.67. click here to view supplementary material
