# Supplementary material for: Teaching psychiatry to medical students in the time of COVID-19: experiences from UK medical schools
Source: BJPsych Bull. 2021 Jul 27:1–10. doi: 10.1192/bjb.2021.67 (PMC8326675; doi:10.1192/bjb.2021.67)
Supplement: Supplementary file 1 [file S205646942100067Xsup001.docx]

**Adaptations to Psychiatry teaching in UK medical schools in response to COVID-19**

Please answer this questionnaire in relation to teaching in the main Psychiatry Rotation at your medical school for the current academic year.

N.B. If Psychiatry is also taught in other parts of the course, then please give details about any changes this year to those parts in the final question.

**Section 1**

Q1. What is the name of the medical school where you are the Psychiatry teaching lead? (required)

Q2. What is your name and email address? (optional)

**Section 2: Changes to teaching delivery**

Q3. Has the proportion of time spent by students learning using the following approaches increased, decreased or stayed the same?

|  | Increased | Stayed the same | Decreased | N/A (approach not used) |
| --- | --- | --- | --- | --- |
| Lectures |  |  |  |  |
| Small group teaching e.g. seminars, tutorials, problem-based learning |  |  |  |  |
| Clinical skills sessions, role-play or simulation |  |  |  |  |
| Online tasks e.g. videos, virtual cases, e-learning modules |  |  |  |  |
| Sessions officially designated for self-directed learning |  |  |  |  |
| Independent project work |  |  |  |  |
| Workplace learning e.g. clinical placements |  |  |  |  |

Q4. Before the COVID-19 pandemic, how were the following approaches to teaching delivered?

|  | Face-to-face only | Mixture of face-to-face and online | Online only | N/A (approach not used) |
| --- | --- | --- | --- | --- |
| Lectures |  |  |  |  |
| Small group teaching e.g. seminars, tutorials, problem-based learning |  |  |  |  |
| Clinical skills sessions, role-play or simulation |  |  |  |  |
| Workplace learning e.g. clinical placements |  |  |  |  |

Q5. How are the following approaches to teaching delivered during the current academic year?

|  | Face-to-face only | Mixture of face-to-face and online | Online only | N/A (approach not used) |
| --- | --- | --- | --- | --- |
| Lectures |  |  |  |  |
| Small group teaching e.g. seminars, tutorials, problem-based learning |  |  |  |  |
| Clinical skills sessions, role-play or simulation |  |  |  |  |
| Workplace learning e.g. clinical placements |  |  |  |  |

Q6. Has the content of the Psychiatry curriculum changed?

Yes → Q7

No → Q8

Q7. Please describe the changes to the Psychiatry curriculum.

Q8. Has the amount of teaching in the following subspecialties increased, decreased or stayed the same?

|  | Increased | Stayed the same | Decreased | N/A (no teaching in this subspecialty) |
| --- | --- | --- | --- | --- |
| General adult |  |  |  |  |
| Old age |  |  |  |  |
| Child and adolescent |  |  |  |  |
| Forensic |  |  |  |  |
| Learning disability |  |  |  |  |
| Medical psychotherapy |  |  |  |  |

Q9. What percentage of your online teaching is delivered SYNCHRONOUSLY?

Q10. For online SYNCHRONOUS teaching, which platform(s) do you use? (please select all that apply)

Cisco Webex

Google Meet

Microsoft Teams

Skype

University’s own virtual learning environment e.g. Blackboard, Moodle

Zoom

Other (please state)

Q11. Which existing resources have you used for ASYNCRHONOUS teaching? (please select all that apply)

Recordings of previous content from my medical school

Recordings of previous content from other medical schools

E-learning resources produced by my medical school

E-learning resources obtained through the RCPsych Undergraduate Education Forum

E-learning resources produced by other medical schools, not obtained through the RCPsych Undergraduate Education Forum

E-learning resources in the public domain

Publicly accessible – audio/video podcasts (free)

Publicly accessible – audio/video podcasts (paid)

Other (please state)

Q12. Have you created new resources for ASYNCHRONOUS teaching?

Yes → Q13

No → Q14

Q13. Please describe the new resources that you have created.

Q14. Please write any other comments on changes to teaching delivery here.

**Section 3: Changes to the Psychiatry Rotation, including clinical placements**

Q15. Before the COVID-19 pandemic, how long was the Psychiatry Rotation OVERALL (in weeks)?

Q16. In the current academic year, how long is the Psychiatry Rotation OVERALL (in weeks)?

Q17. Before the COVID-19 pandemic, how long were CLINICAL PLACEMENTS in Psychiatry (in weeks)?

Q18. In the current academic year, how long are CLINICAL PLACEMENTS in Psychiatry (in weeks)?

Q19. In the current academic year, have the number of students placed in the following clinical environments increased, decreased or stayed the same?

|  | Increased | Stayed the same | Decreased | N/A (no students placed in this environment) | I don’t know |
| --- | --- | --- | --- | --- | --- |
| Inpatient wards |  |  |  |  |  |
| Day hospital |  |  |  |  |  |
| Home Treatment Team |  |  |  |  |  |
| Community team |  |  |  |  |  |
| Liaison psychiatry |  |  |  |  |  |

Q20. In the current academic year, have the number of students placed in the following subspecialties increased, decreased or stayed the same?

|  | Increased | Stayed the same | Decreased | N/A (no students placed in this subspecialty) | I don’t know |
| --- | --- | --- | --- | --- | --- |
| General adult |  |  |  |  |  |
| Old age |  |  |  |  |  |
| Child and adolescent |  |  |  |  |  |
| Forensic |  |  |  |  |  |
| Learning disability |  |  |  |  |  |
| Medical psychotherapy |  |  |  |  |  |

Q21. Are tele-conferencing approaches used in clinical placements to allow students to attend the workplace virtually (e.g. virtual ward rounds, telephone or video consultations)?

Yes, in all placements → Q22

Yes, in some placements → Q22

No → Q24

I don’t know → Q24

Q22. Please describe how tele-conferencing approaches have been used in clinical placements.

Q23. How have students responded to the use of tele-conferencing approaches in clinical placements?

Q24. Please write any other comments on changes to the Psychiatry Rotation here.

**Section 4: Changes to assessment**

Q25. Has assessment of the Psychiatry Rotation changed compared to before the COVID-19 pandemic?

Yes → Q26

No → Q27

Q26. How has assessment of the Psychiatry Rotation changed?

Q27. Please write any other comments on changes to assessment here.

**Section 5: Supporting learners**

Q28. Has the Psychiatry education faculty been involved in developing resources or interventions to support learners’ mental health and wellbeing during the COVID-19 pandemic?

Yes → Q29

No → Q30

Q29. Please describe what resources or interventions have been developed.

Q30. Please write any other comments on supporting learners here.

**Section 6: Faculty development**

Q31. Has the faculty received training in adapting teaching in response to COVID-19?

Yes → Q32

No → Q33

Q32. Please describe the training that the faculty has received in adapting teaching in response to COVID-19.

Q33. Has the faculty received training in teaching online?

Yes → Q34

No → Q35

Q34. Please describe the training that the faculty has received in teaching online.

Q35. Has the faculty received additional funding or resources to deliver the Psychiatry teaching this academic year FROM THEIR MEDICAL SCHOOL?

Yes → Q36

No → Q37

Q36. Please describe the additional funding or resources that have been received FROM THE MEDICAL SCHOOL.

Q37. Has the faculty received additional funding or resources to deliver the Psychiatry teaching this academic year FROM THEIR TRUST?

Yes → Q38

No → Q39

Q38. Please describe the additional funding or resources that have been received FROM THE TRUST.

Q39. Please write any other comments on faculty development here.

**Section 7: The impact of adaptations**

Q40. To what extent do you agree or disagree with the following statements?

|  | Strongly agree | Agree | Neither agree nor disagree | Disagree | Strongly disagree |
| --- | --- | --- | --- | --- | --- |
| “Students were involved in adapting the Psychiatry course to COVID-19.” |  |  |  |  |  |
| "Students have had a positive response to the adaptations we have made to the Psychiatry course." |  |  |  |  |  |
| “The COVID-19 pandemic has raised the profile of Psychiatry among medical students.” |  |  |  |  |  |
| "The COVID-19 pandemic will change Psychiatry education for the better." |  |  |  |  |  |

Q41. What do you think works well in delivering the current Psychiatry course?

Q42. What do you think could be improved in delivering the current Psychiatry course?

Q43. What are students positive about regarding the current Psychiatry course?

Q44. What are students critical about regarding the current Psychiatry course?

Q45. Following the COVID-19 pandemic, will the Psychiatry Rotation return to exactly how it was before the pandemic?

Yes → Q47

No → Q46

Q46. What changes will you make to the Psychiatry Rotation after the COVID-19 pandemic?

Q47. If Psychiatry is also taught outside of the main Psychiatry Rotation, please describe adaptations in that teaching in response to COVID-19. and which year group the teaching takes place in.

Q48. Any other comments or suggestions for further study?

Q49. Would you be willing to take part in a telephone or online interview to provide more details?

Yes → Q50

No → End

Q50. Please provide your name and email address (if not provided above).
